# CD74 interacts with CD44 and enhances tumorigenesis and metastasis via RHOA-mediated cofilin phosphorylation in human breast cancer cells

**DOI:** 10.18632/oncotarget.11945

**Published:** 2016-09-10

**Authors:** Zhiyong Liu, Shuzhou Chu, Shun Yao, Yu Li, Songqing Fan, Xiaoyang Sun, Ling Su, Xiangguo Liu

**Affiliations:** ^1^ Shandong University School of Life Sciences, Jinan, China; ^2^ Department of Pathology, The Second Xiangya Hospital of Central South University, Changsha, China

**Keywords:** CD74, CFL1, CD44, RHOA

## Abstract

CD74, also known as Ii, was initially considered to participate primarily in antigen presentation. Subsequent studies have shown that CD74 is highly expressed in various types of tumor cells and has multiple roles in a variety of biological processes. CD74 is thought to promote breast cancer metastasis, but the molecular mechanism remains elusive. In the present study, our results showed that CD74 was more highly expressed on the membrane and in the cytoplasm of breast cancer tissues than in control breast tissues. Consistently, CD74 downregulation reduced MDA-MB-231 cell invasion and migration and suppressed protrusions in breast cancer cells. Moreover, CD74 overexpression promoted the phosphorylation of the actin-severing protein cofilin (CFL1), resulting in actin polymerization in breast cancer cells. CD44 was required for the up-regulation of CFL1 phosphorylation by CD74 because CD44 knockdown downregulated CD74-induced CFL1 phosphorylation, while CD74 overexpression could not rescue CFL1 phosphorylation. Moreover, RHOA is necessary for CFL1 phosphorylation and cell migration induced by CD74 in breast cancer cells. Our findings highlight the critical role of CD74 in breast cancer metastasis. New drugs and antibodies targeting CD74 may be effective strategies for breast cancer therapy.

## INTRODUCTION

Breast cancer is the second most lethal cancer in women in the US, after lung cancer [1, 2]. Cancer cell spread and colonization of distant organs are the major reasons for breast cancer deaths [3, 4]. During the metastatic process, tumor cells escape from their primary location and migrate through the mesenchyme. Metastatic tumors are formed when disseminated cells have extravasated from the vasculature colonize distant organs [5, 6].

CD74, also known as Ii, is the invariant chain of major histocompatibility complex (MHC) class II. CD74 was initially thought to participate primarily in antigen presentation. In the endoplasmic reticulum (ER), CD74 forms a homotrimeric structure and interacts with MHC class II, which can prevent the incorrect interaction of endogenous peptides with MHC class II [7, 8]. In endocytic compartments, CD74 is subjected to proteolytic cleavage, and MHC class II can interact with exogenous immunologic peptides. Finally, the complex is transported to the surface of the antigen-presenting cell and presents exogenous peptides to CD4 (+) T cells [8].

CD74 is believed to be involved in tumor metastasis. CD74 has a low expression level in normal epithelial cells but is highly expressed in a variety of tumor cells, including breast cancer cells [1]. Oncogenic fusion genes with *CD74* have been reported to be involved in cancer development, e.g., *CD74-NRG1* and *CD74-ROS1* fusion genes are associated with lung malignancy [9, 10]. In clear cell-renal cell carcinoma, the tumor grade was related to CD74 expression, and downregulation of CD74 induced cell cycle arrest and apoptosis, while cell proliferation and invasion were suppressed [11]. Moreover, tumor invasion in pancreatic cancer and thyroid carcinoma were reported to be associated with CD74 [12, 13]. CD74 is also a receptor of macrophage migration inhibitory factor (MIF), which can activate signaling pathways for the immune response, cell proliferation and survival [14–16]. The hyaluronan receptor, CD44, interacts with CD74 and is critical for MIF-induced cell signaling pathways in B cells [17].

Actin cytoskeleton reorganization is regulated by a series of actin-binding proteins, such as CFL1, actin-related protein 2/3 (ARP2/3), PFN1, capping protein, etc. And CFL1 is reported to be involved in cell motility and metastasis [18]. CFL1 severs and disassembles actin filaments to regulate actin cytoskeleton rearrangement. The actin-severing activity of CFL1 is suppressed by phosphorylation at Ser3, and Ser3 is essential for CFL1 to respond to upstream stimuli [18]. Hence, upstream regulators such as the Rho family of small GTPases, play a critical role in regulating actin cytoskeleton reorganization and related cell motility [19].

The Rho family of small GTPases, including RHOA, RAC, and CDC42, are members of Ras superfamily of small GTP-binding proteins. Rho GTPases have two phases, GDP-bound state (inactive) and GTP-bound state (active) [20]. Activation of Rho GTPases stimulates downstream effectors and regulates CFL1-dependent actin cytoskeleton rearrangement [5, 21]. Therefore, Rho GTPases are associated with multiple cellular functions, including cell migration and invasion [22].

In this study, our results show that CD74 and CD44 coordinately promote actin polymerization via RHOA-mediated CFL1 phosphorylation, enhancing tumor cell metastasis.

## RESULTS

### CD74 expression correlated with clinical stages and lymph node metastasis in breast cancer

**Figure 1:**
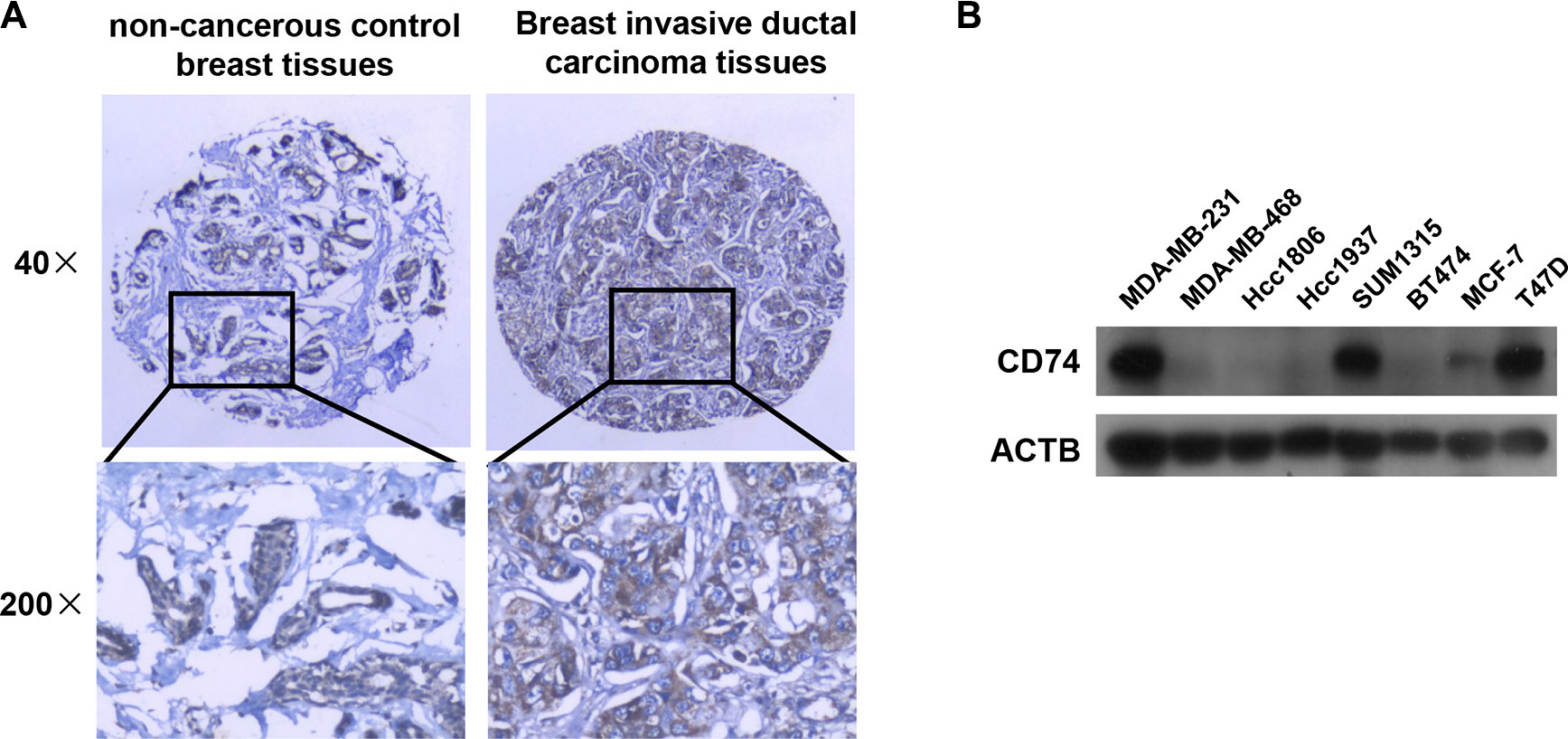
CD74 distribution in tissues and expression in breast cancer cell lines (**A**) Representative immunohistochemical staining of CD74 in non-cancerous control breast tissues (NCBT) and breast invasive ductal carcinoma (BIDC) tissues. (**B**) The CD74 protein level was examined in 8 breast cancer cell lines by western blotting.

Immunohistochemistry (IHC) was used to investigate the expression level of CD74 in NCBT and BIDC tissues. The IHC results showed that CD74 was highly expressed on the membrane and in the cytoplasm of breast cancer tissues compared with control breast tissues (Figure 1A). To clarify the association between the expression of CD74 protein and the clinicopathological features of BIDC, 189 BIDC tissues and 40 NCBT tissues were involved in our study. The patient characteristics are shown in Supplementary Table S1. Chi-square analysis indicated that elevated expression of CD74 was associated with breast cancer; the CD74 expression level was increased in 143 of 189 (75.7%) cases with BIDC (Supplementary Table S2). Multivariate logistic regression analysis of lymph node metastasis (LNM) factors in BIDC patients revealed that LNM was associated with the clinical stage and CD74 expression level (Supplementary Table S3). Chi-square analysis of the relationship between CD74 and clinicopathological features also indicated that CD74 was significantly associated with clinical stage and LNM; an increased percentage of cases with LNM had high CD74 expression levels (80.0%; Supplementary Table S4). Taking these data together, we found that CD74 was highly expressed in BIDC, and the CD74 expression level was associated with both clinical stage and lymph node metastasis.

### Different expression levels of CD74 in breast cancer cell lines

High expression of CD74 has been reported to be associated with a variety of carcinomas [17, 23], including breast cancer. Different expression levels of CD74 were detected in different breast cancer cell lines by western blotting (Figure 1B). The results showed that CD74 was highly expressed in MDA-MB-231, SUM1315 and T47D cell lines, while MDA-MB-468, Hcc1806, Hcc1937, BT474 and MCF-7 cell lines had a lower CD74 expression level. T47D and the highly metastatic cell line MDA-MB-231 were selected for the subsequent suppression of CD74 expression, and overexpression of CD74 was conducted in Hcc1806 cells and the less metastatic cell line MCF-7.

### Downregulation of CD74 suppressed the invasion and migration of MDA-MB-231 cells

**Figure 2:**
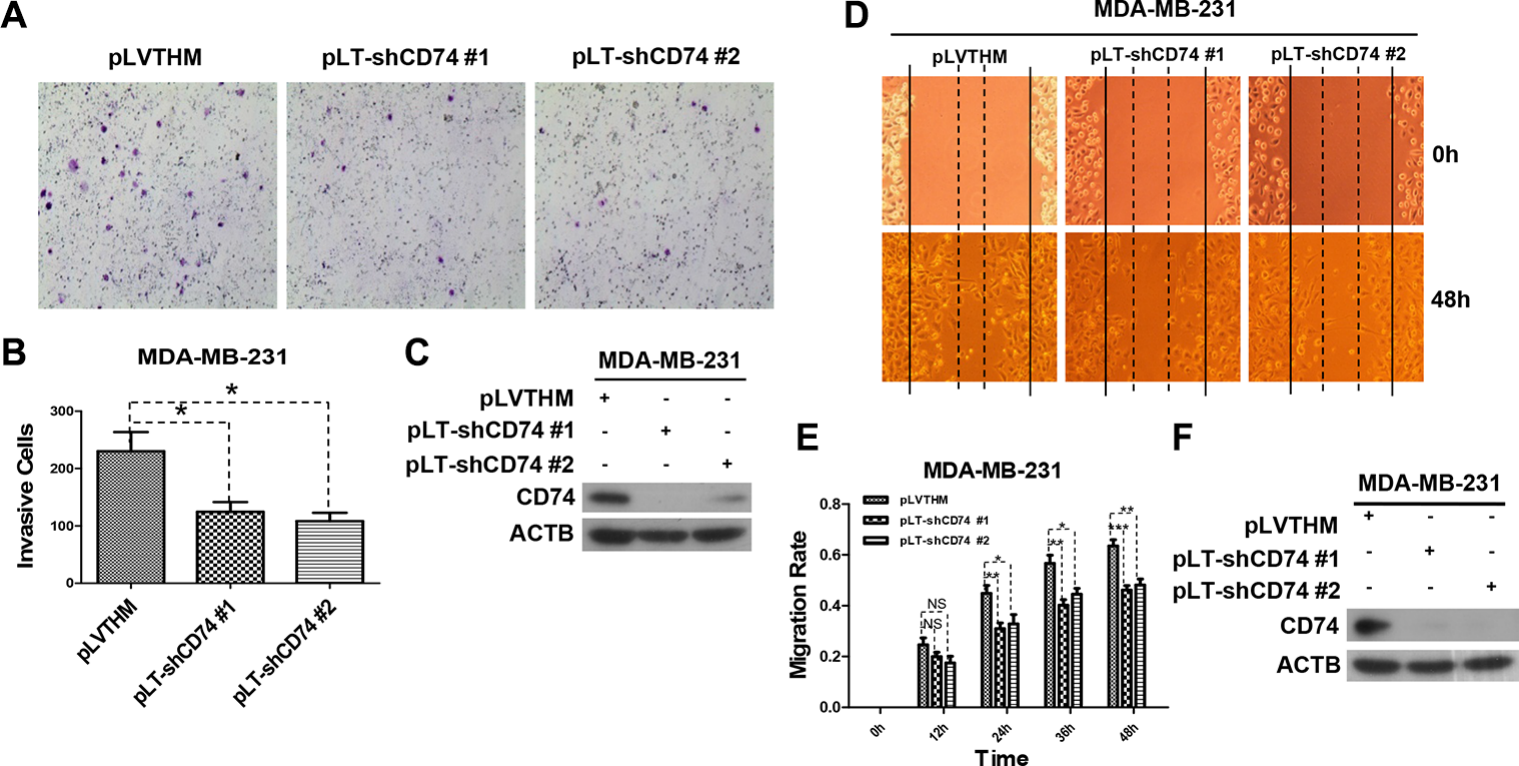
CD74 knockdown inhibited cell migration (**A**) MDA-MB-231 cells were transfected with pLVTHM (control) or pLT-shCD74 vectors (pLT-shCD74 #1 and pLT-shCD74 #2) for 48 h. Then, the cells were resuspended and counted, and 4 × 10^5^ cells in 500 μl of serum-free DMEM 3.7 medium were added to the upper well, while the lower well contained NIH3T3 conditioned medium (600 μl). The cells were incubated in a humidified culture incubator for 48 h. Representative images of cell invasion are shown. (**B**) The invading cells were counted. The error bars represent the SD, *P < 0.05. (**C**) MDA-MB-231 cells in each group were lysed after transfection, and the CD74 protein level was detected by western blotting. (**D**) MDA-MB-231 cells were transfected with pLVTHM or pLT-shCD74 plasmids, and a wound-healing scratch assay was conducted when the cells had grown to confluence. The images represent experiments at 0 and 48 h for each group. (**E**) The average width was recorded every 12 h, and differences between the treatment groups were analyzed by *t*-test. The error bars represent the SD, *P < 0.05; **P < 0.01; ***P < 0.001; ns, not significant. (**F**) Cells in different groups were collected and lysed at the end of a wound-healing scratch assay, and CD74 downregulation was detected by western blot.

CD74 shRNAs were designed as described in the Materials and Methods, and shRNAs were subcloned into the pLVTHM vector to construct the recombinant vectors pLT-shCD74 #1 and pLT-shCD74 #2. CD74 shRNA-containing vectors were transfected into MDA-MB-231 cells with a high endogenous CD74 level. Western blot results showed that CD74 expression was effectively suppressed by CD74 shRNAs (Supplementary Figure S1A). MDA-MB-231 is a highly metastatic cell line with high expression of CD74. A Matrigel invasion assay showed that MDA-MB-231 cell migration from the upper well to the lower well was markedly inhibited by CD74 downregulation (Figure 2A–2C). A wound-healing scratch assay also showed that the migration ability of MDA-MB-231 cells was significantly suppressed by inhibiting CD74 expression (Figure 2D–2F). Taken together, our results suggest that the metastatic capacity of MDA-MB-231 cells is suppressed by the inhibition of CD74 expression.

### Cell protrusion formation positively correlated with CD74 expression

**Figure 3:**
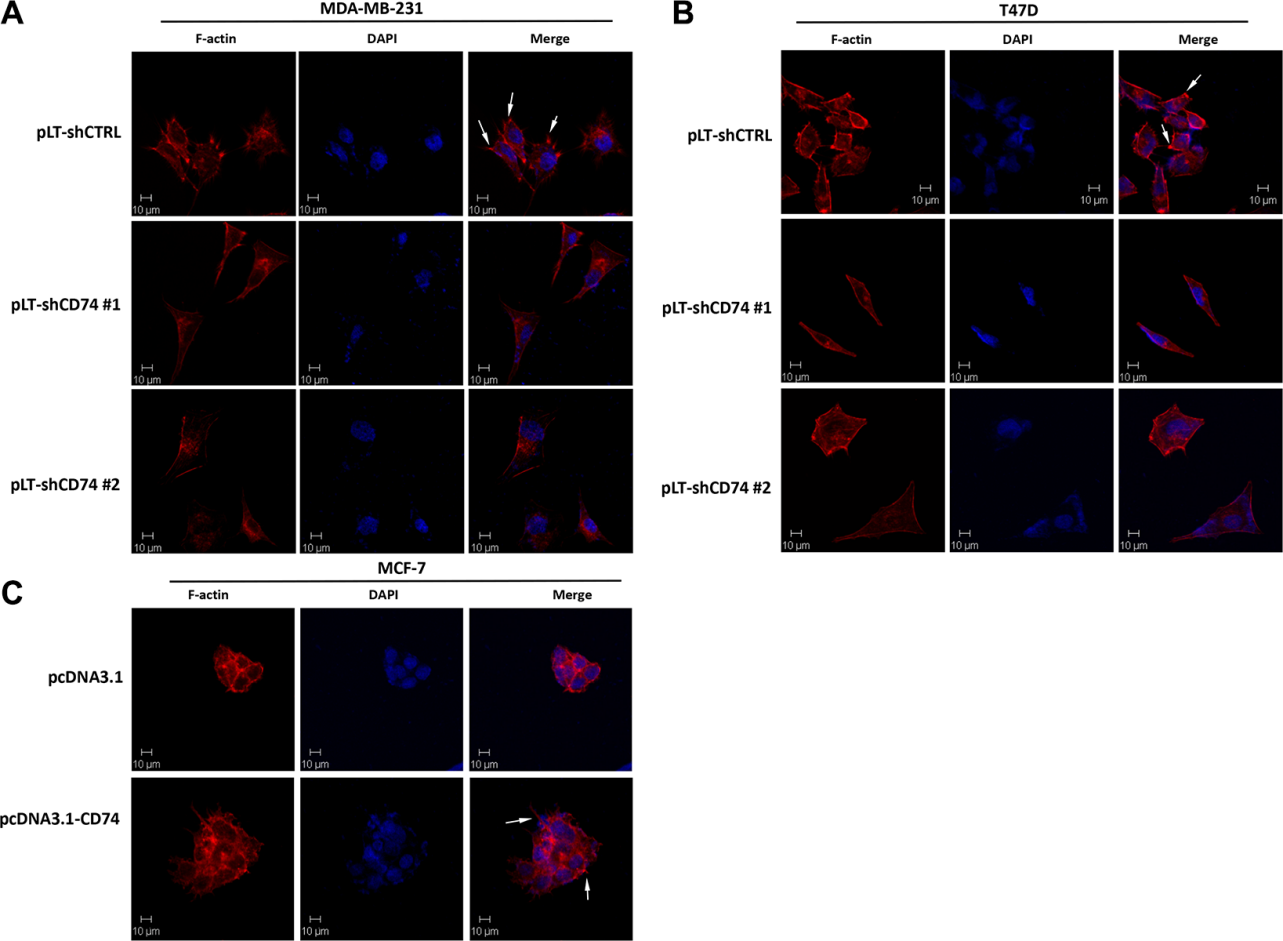
CD74 promoted protrusion formation in breast cancer cells (**A**) MDA-MB-231 cells were transfected with pLVTHM or pLT-shCD74 plasmids for 24 h, and then the cells were reseeded onto adhesive microscope slides, F-actin was stained with TRITC-conjugated phalloidin (red), and the nuclei were stained with DAPI (blue). (**B**) T47D cells were treated similar to MDA-MB-231 cells, and representative images are shown as indicated. (**C**) MCF-7 cells were transfected with pcDNA3.1 or pcDNA3.1-CD74, and the cells were reseeded onto adhesive microscope slides after 24 h of transfection. F-actin was stained with TRITC-conjugated phalloidin (red), and the nuclei were visualized with DAPI (blue). Arrows stand for the typical protrusions.

Cell metastasis is associated with pseudopodium formation. Thus, CD74-regulated cell metastatic capacity may have a relationship with cell protrusion and pseudopodium formation. pLT-shCD74 vectors were transfected into the CD74 highly expressing MDA-MB-231 and T47D cell lines, and cell morphology was visualized with a laser scanning confocal microscope. The imaging data showed that CD74-inhibited cells exhibited a smoother cell edge both in MDA-MB-231 (Figure 3A) and T47D cells (Figure 3B). CD74 shRNA-transfected cells had fewer filopodia-like protrusions than the control group. More protrusion were formed when CD74 was overexpressed in MCF-7 cells, which had a low endogenous CD74 level (Figure 3C). These data suggest that CD74 expression positively correlated with cell protrusion and pseudopodium formation.

### CD74 promotes CFL1 phosphorylation

**Figure 4:**
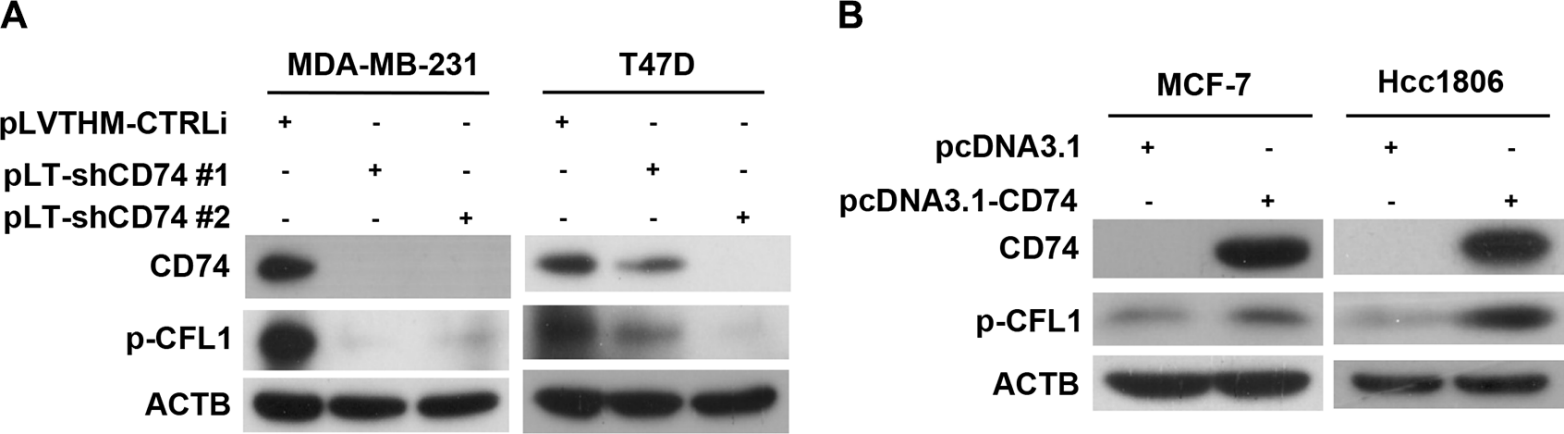
CD74 increased CFL1 phosphorylation in breast cancer cells (**A**) MDA-MB-231 and T47D cells were transfected with CD74 shRNA-containing vectors for 48 h, and then, CFL1 phosphorylation was detected by western blot. (**B**) Overexpression of CD74 by transfection of the pcDNA3.1-CD74 vector in MCF-7 and Hcc1806 cells. Western blot analysis was used for the detection of CFL1 phosphorylation.

CFL1 is an actin-binding protein, and net actin polymerization and depolymerization are regulated by the actin-severing activity of CFL1 [24]. CFL1 switches between the phosphorylated and the non-phosphorylated form, and p-CFL1 loses actin-severing activity leading to actin polymerization. To investigate whether CFL1 participated in CD74-dependent cell migration, CD74 was silenced in MDA-MB-231 and T47D cells. The results showed that CD74 inhibition led to p-CFL1 downregulation (Figure 4A). Furthermore, CD74 overexpression was performed in MCF-7 and Hcc1806 cell lines. As expected, the p-CFL1 level was upregulated (Figure 4B). Taking these findings together, our study suggests that CD74 promotes CFL1 phosphorylation in breast cancer cells.

### CD44 plays a critical role in CD74-dependent CFL1 phosphorylation

The hyaluronan receptor CD44 has been reported to be associated with a series of cellular behaviors, including cell invasion and migration. Shi *et al*. reported that CD44 interacted with CD74 in COS-7/M6 cell lines and transduced MIF-induced ERK1 and ERK2 activation [25]. To investigate whether CD74 and CD44 interact with each other in breast cancer cell lines, immunofluorescence was conducted in MDA-MB-231 and T47D cell lines. AlexaFluor 568 dye-stained CD74 (red) merged with AlexaFluor 488 dye-stained CD44 (green); the yellow region showed the areas where CD74 interacted with CD44 (Supplementary Figure S2A). Moreover, an immunoprecipitation (IP) assay was used to confirm the interaction between CD74 and CD44. Flag-CD74 was transfected into MDA-MB-231 cells, and CD44 was co-immunoprecipitated with an anti-Flag antibody. As expected, we obtained the same result in T47D cell lines (Supplementary Figure S2B). In addition, the lysates of MDA-MB-231 cells were used to detect the endogenous interaction between CD74 and CD44. The results showed that CD74 was co-immunoprecipitated with an anti-CD44 antibody (Supplementary Figure S2C). These data suggest that CD74 interactes with CD44 in breast cancer cell lines.

**Figure 5:**
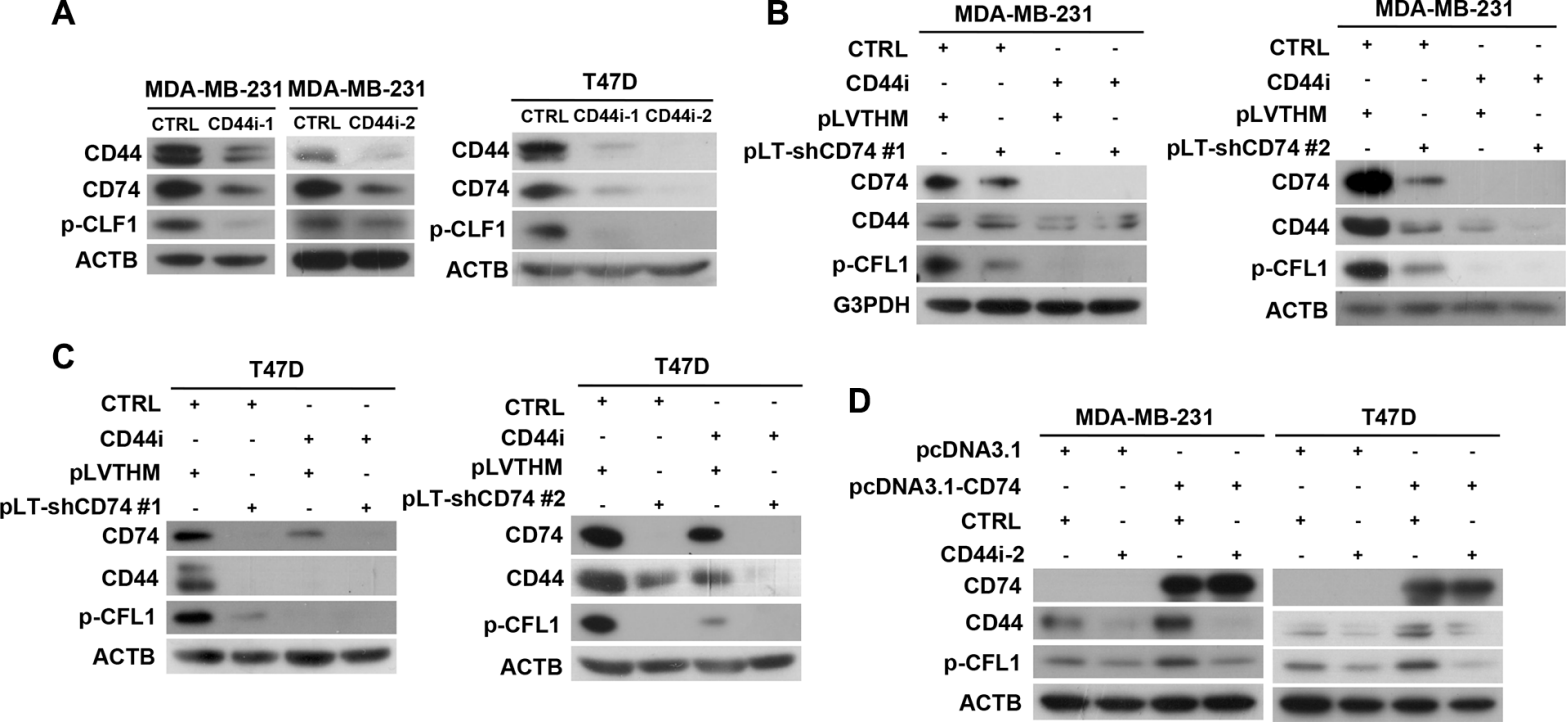
CFL1 phosphorylation was co-regulated by CD74 and CD44 (**A**) CD44 was silenced by CD44-specific siRNA (CD44i-1 or CD44i-2) in MDA-MB-231 and T47D cells, respectively. Western blot showing CFL1 phosphorylation. (**B**) Double knockdown of CD74 and CD44 was performed in MDA-MB-231 cells. CD74 knockdown was conducted by transfecting cells with a CD74 shRNA-containing plasmid (pLT-shCD74 #1 or pLT-shCD74 #2), and CD44 knockdown was conducted by transfecting the cells with CD44-specific siRNA. The western blot shows CFL1 phosphorylation. (**C**) Double knockdown of CD74 and CD44 in T47D cells was performed as described above. (**D**) CD74 overexpression and CD44 knockdown were performed in MD-MB-231 and T47D cells, respectively. p-CLF1 was detected by western blot.

To investigate whether CD44 is required for CD74-dependent p-CFL1 regulation, CD44 was silenced by CD44-specific siRNAs. Western blot results showed that inhibition of CD44 expression led to the suppression of the CD74 protein level, and the CD74-dependent CFL1 phosphorylation level was downregulated. Consistent results were obtained both in MDA-MB-231 and T47D cell lines (Figure 5A). To further determine the relationship between CD74, CD44 and p-CFL1, inhibition of CD74 and/or CD44 was performed in MDA-MB-231 cells, in which CD74 and CD44 are expressed simultaneously. Western blot results suggested that CFL1 phosphorylation was suppressed when either CD74 or CD44 was downregulated. The CFL1 phosphorylation level was further reduced when CD74 and CD44 were both suppressed in MDA-MB-231 cells (Figure 5B). Consistent results were obtained in T47D cells (Figure 5C). In the rescue experiment, CD44 knockdown induced a reduction in the p-CFL1 level that could not be restored completely by overexpression of CD74 (Figure 5D), suggesting that CD44 was required for CD74-regulated CFL1 phosphorylation. Taken together, our results indicate that CD44 was involved in CD74-dependent CFL1 phosphorylation regulation, and CFL1 phosphorylation was enhanced by CD74 through the CD44 pathway.

To elucidate the effect of CD74 and CD44 on cell mobility, TRITC-conjugated phalloidin was used to stain F-actin. Cell morphology was visualized with a laser scanning confocal microscope. The imaging results showed that compared with the control group, MDA-MB-231 cells with CD74 or CD44 only knockdown exhibited a smoother cell edge, with fewer protrusions than the control group. The CD74 and CD44 double-knockdown group also showed fewer protrusions compared with control group, but the group exhibited little further decrease in cell pseudopodium formation; this result might be because the inhibition of CD44 had inhibited the majority of cell pseudopodium formation (Supplementary Figure S3A). Consistent results were obtained in T47D cells (Supplementary Figure S3B).

Taken together, our results suggest that CD44 was involved in CD74-dependent CFL1 phosphorylation regulation, and CD74 and CD44 inhibition coordinately suppressed CFL1 phosphorylation and cell pseudopodium formation.

### CD74 promotes CFL1 phosphorylation through RHOA

**Figure 6:**
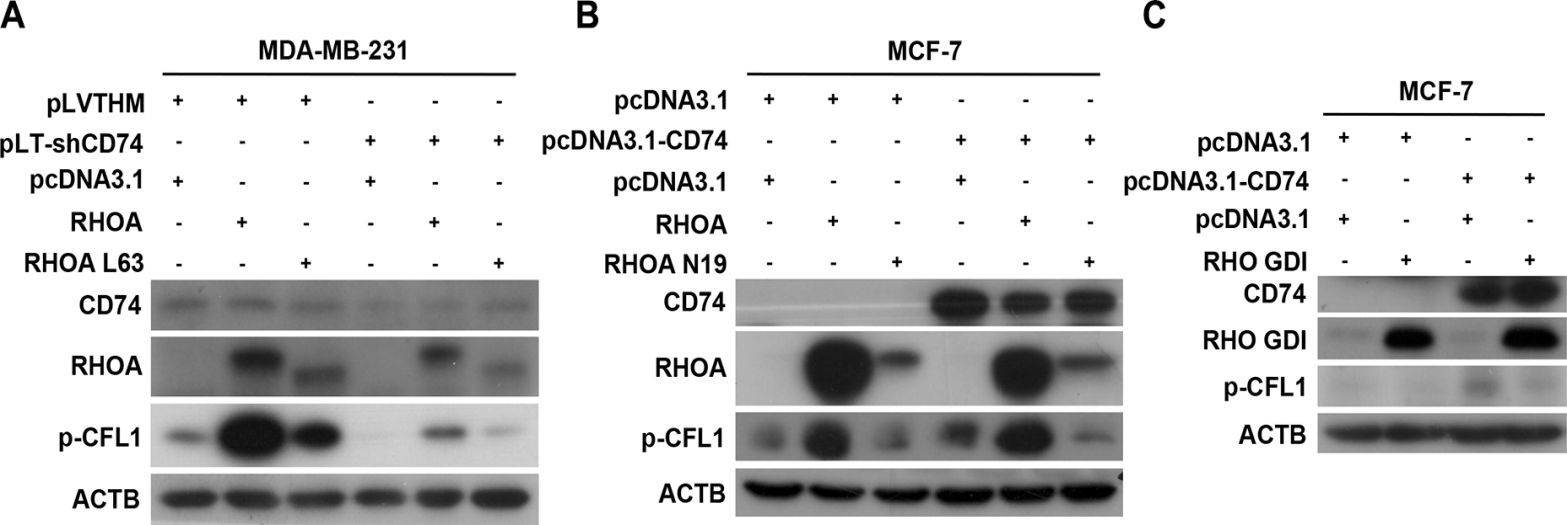
CD74 enhanced CFL1 phosphorylation through RHOA (**A**) MDA-MB-231 cells were co-transfected with a CD74 shRNA-containing plasmid and a RHOA L63 (Constitutively Active) vector as indicated, and the proteins were immunoblotted with p-CFL1 antibody. (**B**) MCF-7 cells were co-transfected with pcDNA3.1-CD74 and RHOA N19 (Dominant Negative) plasmids as indicated, and RHOA activity-dependent CFL1 phosphorylation was detected by western blot assay. (**C**) MCF-7 cells were co-transfected with pcDNA3.1-CD74 and RHO GDI, and CFL1 phosphorylation was detected by immunoblot assay.

To examine which Rho GTPases are involved in CD74-regulated CFL1 phosphorylation, four plasmids were used: RHOA L63 (constitutively active); RHOA N19 (dominant negative); RAC N17 (dominant negative); and CDC42 N17 (dominant negative). Inhibition of CD74 expression and overexpression of RHOA or RHOA L63 were performed in MDA-MB-231 cells. The western blot results showed that CD74 suppression induced p-CFL1 downregulation, but the p-CFL1 level was rescued when RHOA or RHOA L63 was overexpressed (Figure 6A). Overexpression of CD74, RHOA and RHOA N19 in MCF-7 cells suggested that CD74 upregulation promoted CFL1 phosphorylation, and RHOA overexpression further upregulated p-CFL1, but p-CFL1 was significantly inhibited when RHOA N19 was expressed (Figure 6B). These data imply that CD74 regulates CFL1 phosphorylation through RHOA. To further confirm this possibility, two plasmids containing RHO GDI, a RHO activation inhibitor, and CD74 were co-transfected into MCF-7 cells. RHO GDI markedly inhibited the CD74 overexpression-induced p-CFL1 increase (Figure 6C). RAC N17 or CDC42 N17 co-transfected with CD74 did not suppress the CD74-induced upregulation of CFL1 phosphorylation (Supplementary Figure S4A and S4B), and western blot results suggested that RAC and CDC42 were minimally involved in CD74-dependent CFL1 phosphorylation.

Simultaneous knockdown of CD74 and overexpression of RHOA L63 were performed in MDA-MB-231 cells. A wound-healing scratch assay suggested that RHOA L63 could rescue the CD74 knockdown-induced reduction in cell migration (Supplementary Figure S5A–S5C). Co-transfection of the CD74 and RHOA N19 plasmids were conducted in MCF-7 cells. The wound-healing scratch assay showed that RHOA N19 inhibited CD74 upregulation-promoted cell migration (Supplementary Figure S5D–S5F). Furthermore, laser scanning confocal images showed that RHOA N19 suppressed CD74 overexpression-promoted cell protrusion formation (Supplementary Figure S5G). Taken together, our data suggest that CD74 promotes CFL1 phosphorylation and cell migration through the RHOA pathway.

### Downregulation of CD74 suppressed MDA-MB-231 xenograft growth *in vivo*

**Figure 7:**
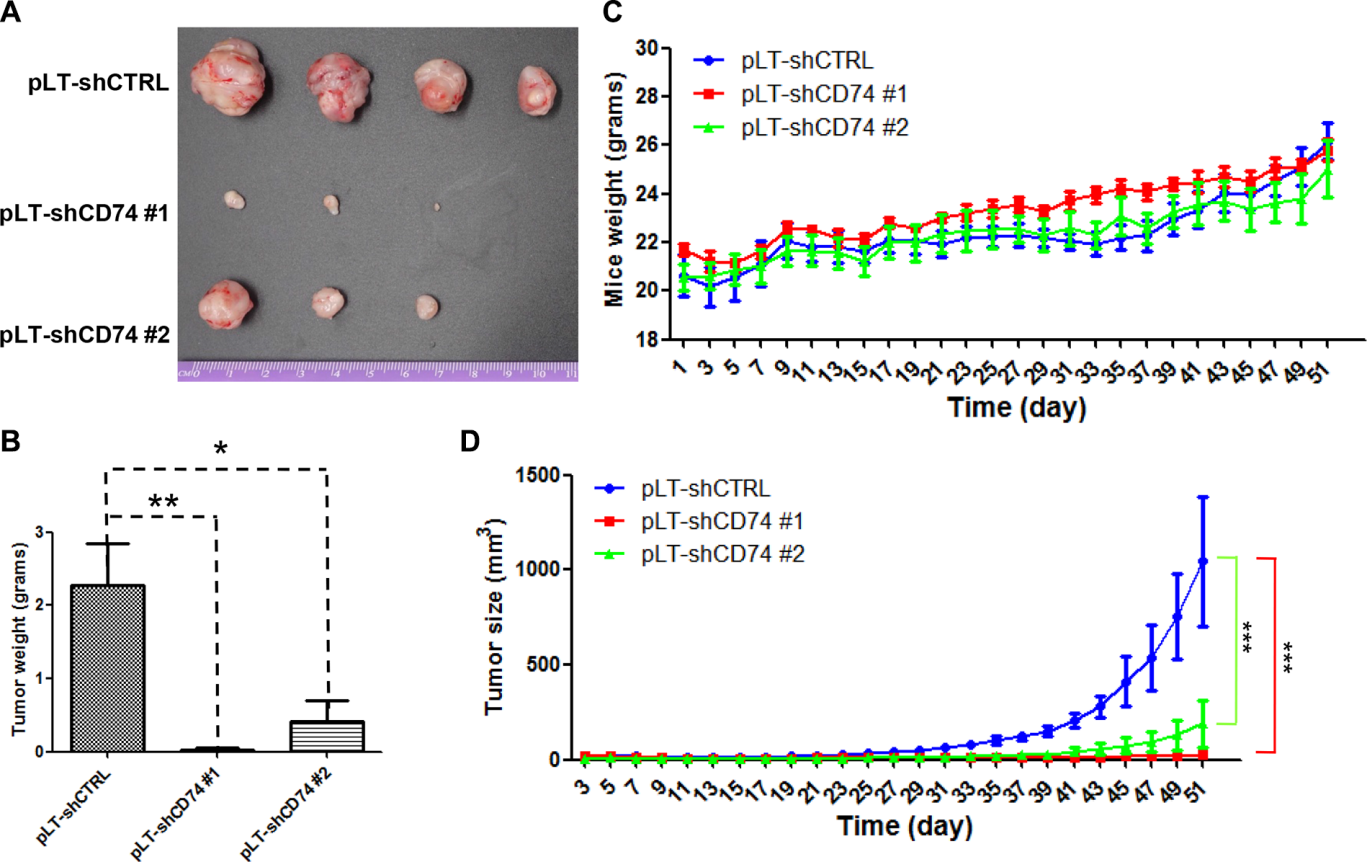
CD74 knockdown inhibits breast cancer tumor growth *in vivo* (**A**) Athymic nu/nu mice were injected with control cells or stably CD74-silenced cells as indicated. After 51 days, the mice were sacrificed, and the tumors in each group are shown. (**B**) Tumor weights were calculated. The error bars represent the SD, *P < 0.05, and **P < 0.01. (**C**) The weight curves of the three groups of mice are shown as indicated. (**D**) The tumor sizes in the three groups of nude mice were measured every two days. ***P < 0.001.

To evaluate whether the tumor growth of MDA-MB-231 cells is regulated by CD74 *in vivo*, MDA-MB-231 pLT-shCD74 #1 and MDA-MB-231 pLT-shCD74 #2 cells and their corresponding control cells, MDA-MB-231 pLT-shCTRL, were injected into the mammary fat pads of athymic mice. The CD74 levels of the three MDA-MB-231 cell lines were detected by western blotting (Supplementary Figure S1B). As shown in Figure 7, the MDA-MB-231 pLT-shCD74 xenografts grew substantially more slowly than the control group, and thus, the tumorigenic potential of MDA-MB-231 cells was reduced (Figure 7A). A case of liver metastasis was found in the control MDA-MB-231 pLT-shCTRL group. However, as shown in Figure 7A, each of the MDA-MB-231 pLT-shCD74 experimental groups had mice without tumor growth. The tumor weights were reduced significantly in MDA-MB-231 pLT-shCD74 xenografts compared with the control group (Figure 7B). There was no significant difference in mouse weight among the three groups (Figure 7C). Tumor growth curves for the pLT-shCTRL, pLT-shCD74 #1 and pLT-shCD74 #1 groups are shown in Figure 7D. A remarkable reduction of tumor growth was found in the MDA-MB-231 pLT-shCD74 xenograft groups.

## DISCUSSION

Our IHC data showed that CD74 was highly expressed in breast cancer tissues. The analysis of clinical data indicated that CD74 expression correlated with clinical stages and lymph node metastasis in breast cancer. To evaluate the role of CD74 in breast cancer metastasis, CD74 was silenced in MDA-MB-231 cells, and our results showed that CD74 downregulation induced a significant reduction in cell invasion and migration. Our confocal results also indicated that CD74 silencing suppressed cell protrusions formation in MDA-MB-231 and T47D cells; smoother cell edges were observed in these cells. When CD74 was overexpressed in MCF-7 cells, more cell protrusions were detected. Greenwood *et al*. showed that STAT1 and CD74 are associated with breast cancer metastasis, and our findings are consistent with their study [23].

Although CFL1 was reported to be positively associated with cell metastasis and pseudopodium formation [26], Bravo-Cordero *et al*. reported that CFL1 induced actin polymerization and depolymerization dependent on the concentration of G-actin [18]. It was also shown that cell invasiveness was determined by the LIMK1/CFL1 rate. Ohashi *et al*. suggested that the downregulation of p-CFL1 reduced cell invasion and migration [19]. Our results indicated that the inhibition of CD74 induced a decrease of p-CFL1 in MDA-MB-231 and T47D cells. As expected, the p-CFL1 level was increased when CD74 was overexpressed in MCF-7 and Hcc1806 cells. Taken together, our results suggested that CD74-dependent p-CFL1 upregulation enhanced breast cell metastasis.

CD44 was reported to regulate breast cancer invasion and migration. Shi *et al*. indicated that CD44 interacted with CD74 in COS-7/M6 cell lines and transduced MIF-induced signaling [25]. Our data showed that CD74 interacted with CD44 in breast cancer cells. Inhibition of CD44 induced a decrease in p-CFL1, which could not be restored by the overexpression of CD74. CFL1 phosphorylation was co-regulated by CD74 and CD44. CD44 also has an effect on cell morphology, and the breast cancer cells with CD74 or/and CD44 knockdown exhibited a smoother cell edge with fewer protrusions.

Rho family small GTPases, including RHOA, RAC, and CDC42, stimulate downstream effectors to regulate CFL1-dependent actin cytoskeleton rearrangement. Our findings showed that RHOA was involved in CD74-dependent p-CFL1 regulation. Furthermore, a wound-healing scratch assay and confocal images confirmed the role of RHOA in CD74-regulated CFL1 phosphorylation.

In the *in vivo* experiment, we show that downregulation of CD74 suppressed MDA-MB-231 xenograft growth. According to our IHC data, CD74 is a marker of breast cancer metastasis. In addition, CD74 interacts with CD44 which is highly expressed in breast cancer stem cells (CSC) [27], hinting that CD74 may play a critical role in generation and maintenance of cancer stem cells (CSC) via regulating the function of CD44. It is regarded that cancer stem cells are responsible for the tumor growth and metastasis *in vivo*. Therefore, it is reasonable that CD74 knockdown suppresses CSC via CD44, resulting in tumor growth inhibition in the xenograft model. However, further study is required to test this hypothesis.

In summary, we demonstrate that CD74 promotes breast cancer cell metastasis, that CD74 and CD44 coordinately upregulate CFL1 phosphorylation through RHOA pathway, and that the repression of CD74 expression suppresses xenograft growth *in vivo*. Because CD74 plays a critical role in the immune response, CD74 may affect tumorigenesis through both actin rearrangement and the immune response. Our findings uncover the molecular mechanism by which CD74 promotes breast cancer metastasis. Powerful drugs and antibodies targeting CD74 may therefore be an effective novel strategy for breast cancer therapy.

## MATERIALS AND METHODS

### Patients and tissue specimens

In this study, 189 patients who had confirmed breast cancer diagnoses and had undergone radiotherapy and/or chemotherapy in the Second Xiangya Hospital of Central South University (Changsha, China) were included. The patients, with a mean age of 45.54 years (range, 24–80 years), were treated between 2002 and 2012. The patients and their relatives provided informed consent, and the tissue specimens were obtained following protocols approved by the Second Xiangya Hospital of Central South University Ethics Review Board. The characteristics of the patients are described in Supplementary Table S1.

### Immunohistochemistry and score

CD74 staining was conducted as follows. CD74 specimens were deparaffinized and rehydrated, and antigen retrieval was performed in a microwave oven at 750 Watts for 30 min. To block endogenous peroxidase activity, the specimens were incubated in methanol containing 0.3% H_2_O_2_ at 37°C for 30 min. Then, to avoid non-specific binding, the samples were incubated with preimmune serum at room temperature for 30 min. Next, the sections were incubated with a CD74-specific primary antibody at 4°C overnight. Following washes with phosphate-buffered saline (PBS) and incubation with a labeled polymer-HRP for 30 min, 3, 3-diaminobenzidine tetrachloride (DAB) was applied to initiate the colorimetric reaction. All sections were then counterstained in hematoxylin.

A semi-quantitative analysis was performed to evaluate differential expression of CD74 in the in non-cancerous control breast (NCBT) tissues and breast invasive ductal carcinoma (BIDC) tissues as follows. The staining intensity and the extent of staining were used to evaluate CD74 distribution. Immunohistochemical staining of sections was scored at 200 × magnification light microscopy independently by SC and SF, who were blinded to the clinicopathological data. The staining intensity for CD74 was scored as 0 (negative), 1 (weak), 2 (moderate), and 3 (strong). According to the percentage of positive-stained cells, the extent of CD74 staining was scored as 0 (0%), 1 (1–25%), 2 (26–50%), 3 (51–75%), and 4 (76–100%). The sum of the staining intensity and staining extent scores ranged from 0 to 7. An optimal cut-off for CD74 was chosen on the basis of a measure as follows: a staining index score of 0–3 was used to define as low expression and 4–7 indicated high expression of CD74 protein. Agreement between the two evaluators was 95%, and all scoring discrepancies were resolved through discussion between the two evaluators.

### Cell culture

The MDA-MB-231, T47D and MCF-7 cell lines were originally obtained from American Type Culture Collection. The cell lines were recently authenticated using Microread Gene Technology (Beijing, China) by STR analysis. The T47D cell line was maintained in RPMI 1640 (Sigma-Aldrich, R6504) supplemented with 5% (v/v) newborn calf serum (NBCS) (Gibco, 1225590). The MDA-MB-231 and MCF-7 cell lines were cultured in high-glucose Dulbecco's modified Eagle's medium (DMEM) (Sigma-Aldrich, D7777) supplemented with 5% (v/v) fetal bovine serum (FBS) (Bovogen, SFBS). All cells were grown at 37°C in a humidified incubator with 5% CO_2_.

### Antibodies and reagents

Commercially available primary antibodies specific for the following proteins were used: CD74 (Sigma-Aldrich, SAB1402715); CD44 (Santa Cruz, sc-7297); p-CFL1 (Sigma-Aldrich, SAB4300115); ROCK1 (Proteintech, 21850-1-AP); RHOA (CST, #2117P); RAC1 (CST, #2465P); and CDC42 (CST, #2466P). TRITC-phalloidin (P1951) was purchased from Sigma-Aldrich, and the Transcriptor First Strand cDNA Synthesis Kit (04897030001) was obtained from Roche.

### siRNA and shRNA design

The CD44 #1 and CD44 #2 siRNAs target the sequences 5′-GCAGATCGATTTGAATATA-3′ and 5′-GTATGACACATATTGCTTC-3′, respectively. The CD74 1st and CD74 2nd shRNA sequences were designed as follows: 1st-CD74i sense: 5′-CGCGCCCCTTAAGAACACCATGGAGATTCAAGAGATCTCCATGGTGTTCTTAAGTTTTTGGAAAT-3′;

1st-CD74i anti-sense: 5′-CGATTTCCAAAAACTTA AGAAC ACCATGGAGATCTCTTGAATCTCCATGGTG TTCTTAAGGGG-3′;

2nd-CD74i sense: 5′-CGCGCCCCCACCAAGTAT GGCAACATGATTCAAGAGAT CATGTTGCCATAC TTGGTGGTTTTTGGAAAT-3′;

2nd-CD74i anti-sense: 5′-CGATTTC CAAAAAC CACCAAGTATGGCAACATGATCTCTTGAATCATG TTGCCATACT TGGTGGGGG-3′. The siRNAs were synthesized by GenePharma (Shanghai, China), and DNA was synthesized by Sangon Botech (Shanghai, China).

### Plasmids

*CD74* gene was amplified from MDA-MB-231 cDNA and subcloned into a pcDNA3.1 vector to construct a pcDNA3.1-CD74 recombinant expression vector. The synthesized CD74-shRNA (shCD74 #1 and shCD74 #2) and CTRL-shRNA DNAs were annealed and subcloned into the pLVTHM vector to construct the CD74 shRNA-containing recombinant vectors pLT-shCD74 #1 and pLT-shCD74 #2 as well as pLT-shCTRL.

### Western blot analysis

Western blot analysis was performed as previously described [28]. The primary antibodies used were described as above.

### Generation of lentiviruses and construction of stable cell lines

293FT cells were transfected with 1 μg of shRNA-containing plasmids (pLT-shCTRL, pLT-shCD74 #1 or pLT-shCD74 #2), 0.75 μg of the packaging plasmid psPAX2 and 0.25 μg of the envelope plasmid pMD2.G using Lipofectamine 2000 (Invitrogen). The cell culture media was exchanged after transfecting overnight, and the lentivirus supernatant was collected at 48 h and 72 h and stored at −80°C. The MDA-MB-231 cell line was transduced with lentivirus supernatant for 24 h before changing to fresh culture media. Monoclonal cells were screened and identified by green fluorescence and western blotting.

### F-actin staining assay and confocal microscopy

Cells were transfected with plasmids or siRNA, and they were then reseeded onto adhesive microscope slides and incubated at 37°C overnight. The cells were fixed and permeabilized with PHEMO buffer (0.025 M HEPES, 0.068 M PIPES, 0.003 M MgCl2·6H2O, 0.015 M EGTA·Na2, 10% DMSO, pH adjusted to 6.8. Additional reagents were added before use, with a final concentration as follows: 0.05% glutaraldehyde, 0.5% Triton X-100, 3.7% formaldehyde). The cells were blocked in PBS containing 3% BSA for 30 min and then incubated with TRITC-phalloidin (Sigma-Aldrich, 400 ng/ml) for 40 min. The nuclei were stained with DAPI (1 μg/ml). Adhesive microscope slides were visualized using a confocal microscope (ZEISS, LSM700).

### Wound-healing scratch assay

Cells were seeded in a 6-well plate and transfected with siRNA or plasmids. A scratch wound-healing assay was conducted when cells were grown to monolayer confluency. The scratches were made with a p-200 pipette tip across the bottom of the 6-well plates, and the suspended cells were washed gently with PBS. The cells were cultured with medium as described above. Then, 1% FBS was added, and the cells were incubated for 48∼72 h at 37°C in a humidified atmosphere with 5% CO_2_. The sites of the scratch wounds were imaged using a microscope equipped with a digital camera. Images were captured at the indicated time points, and the migration rate was calculated.

### Matrigel invasion assay

A transwell system was used for a Matrigel invasion assay. NIH3T3-conditioned medium was collected after culturing NIH3T3 cells overnight with serum-free DMEM. Cells were seeded in a 6-well plate and transfected with siRNA or plasmids. After 24 h, the transfected cells were suspended and reseeded into the upper well, which was polymerized with Matrigel (25,000 cells in 500 μl of serum-free medium). NIH3T3-conditioned medium (600 μl) was added to the lower well. The cells were incubated for 60 h at 37°C in a humidified atmosphere with 5% CO_2_. Cotton swabs were used to wipe the cells and the Matrigel in the upper well. Invasive cells on the underside of the membrane were stained with crystal violet and counted under a microscope.

### Xenograft studies

Female athymic nu/nu 5-week-old mice were purchased from Vital River Laboratories (Beijing, China). The athymic mice were divided into 3 groups: MDA-MB-231 pLT-shCTRL, MDA-MB-231 pLT-shCD74 #1, and MDA-MB-231 pLT-shCD74 #2. A total of 2 × 10^6^ cells suspended in 100 μl of PBS were injected into the mammary fat pad. The tumor volume was calculated as V = π × (length × width^2^)/6.

### Statistical analyses

A Chi-square test was used to analyze CD74 expression in breast invasive ductal carcinoma (BIDC) and non-cancerous control breast tissue (NCBT) to evaluate the association between the protein expression of CD74 and the clinicopathological features of BIDC. Multivariate logistic regression was used to analyze lymph node metastasis factors in BIDC patients. Clinical data were analyzed with SPSS statistics software. Statistical analysis was expressed as the mean ± standard deviation. Student's *t* test was used for analysis, and *P* < 0.05 was considered significant.

## SUPPLEMENTARY MATERIALS FIGURES AND TABLES


