# Supplementary material for: CD74 interacts with CD44 and enhances tumorigenesis and metastasis via RHOA-mediated cofilin phosphorylation in human breast cancer cells
Source: Oncotarget. 2016 Sep 10;7(42):68303–13. doi: 10.18632/oncotarget.11945 (PMC5356556; doi:10.18632/oncotarget.11945)
Supplement: Supplementary file 1 [file oncotarget-07-68303-s001.pdf]

CD74 interacts with CD44 and enhances tumorigenesis and metastasis via RHOA-mediated cofilin phosphorylation in human breast cancer cells

Supplementary Materials

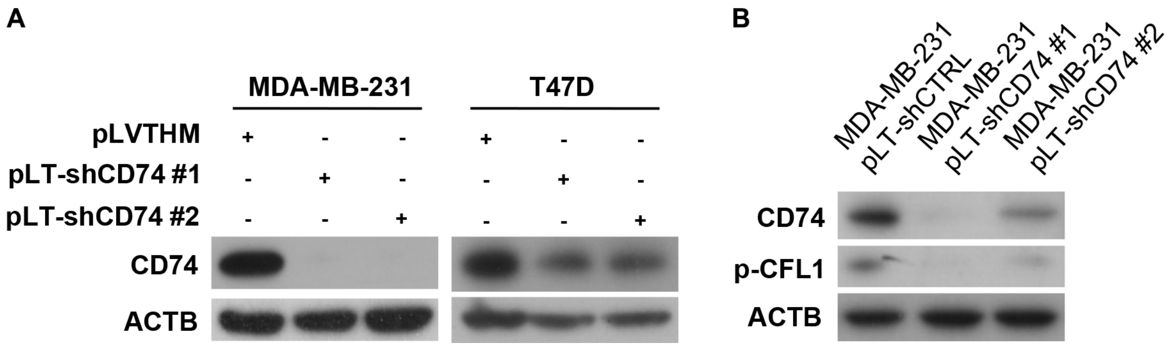

Supplementary Figure S1: Detection of CD74 expression level. (**A**) CD74 was knocked down using CD74 shRNA contained vector in MDA-MB-231 and T47D cells, CD74 level was assayed by western blot. (**B**) CD74 expression level in MDA-MB-231 pLT-shN. **C**., MDA-MB-231 pLT-shCD74 #1 and MDA-MB-231 pLT-shCD74 #2 cell lines were detected by western blot.

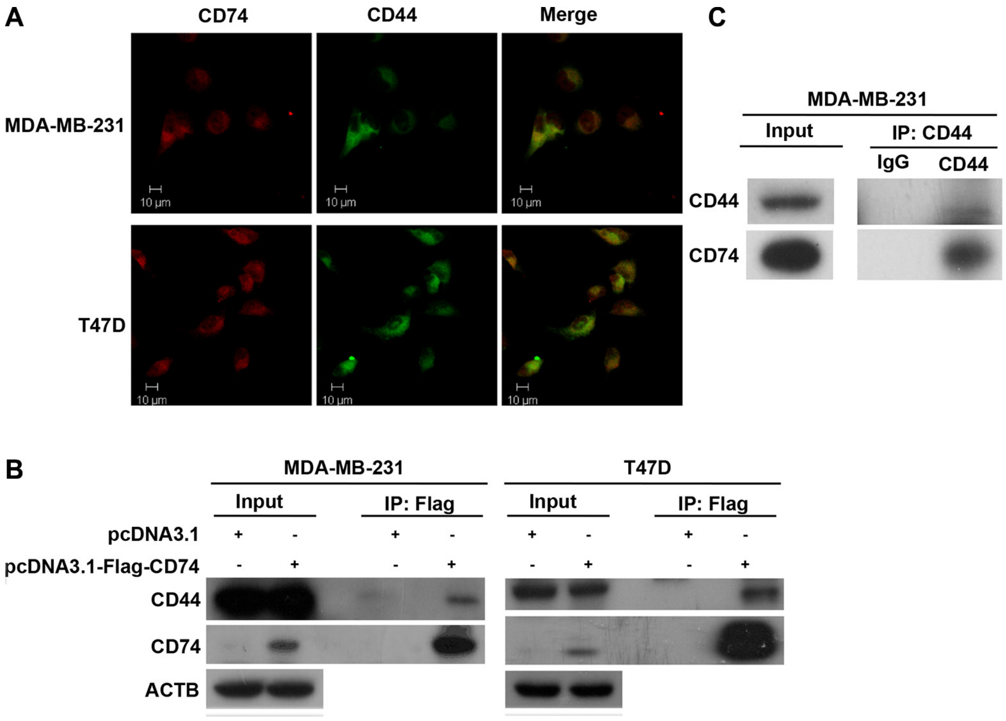

Supplementary Figure S2: CD74 binds to CD44 in human breast cancer cells. (**A**) For immunofluorescence, CD74 was stained with AlexaFluor 568 dye (red), CD44 was stained with AlexaFluor 488 dye (green), yellow staining showed that CD74 colocalized with CD44. (**B**) MDA-MB-231 and T47D cells were transfected with pcDNA3.1 or pcDNA3.1-Flag-CD74, cell lysate was immunoprecipitated with anti-Flag antibody, CD74 and CD44 were detected by western blot. (**C**) For immunoprecipitation, MDA-MB-231 cell lysate was seperated into two group and incubated with IgG antibody and CD44 antibody at 4°C for 1 h, respectively. Then, cell lysate incubated with protein G at 4°C overnight, CD74 and CD44 protein were detected by western blot.

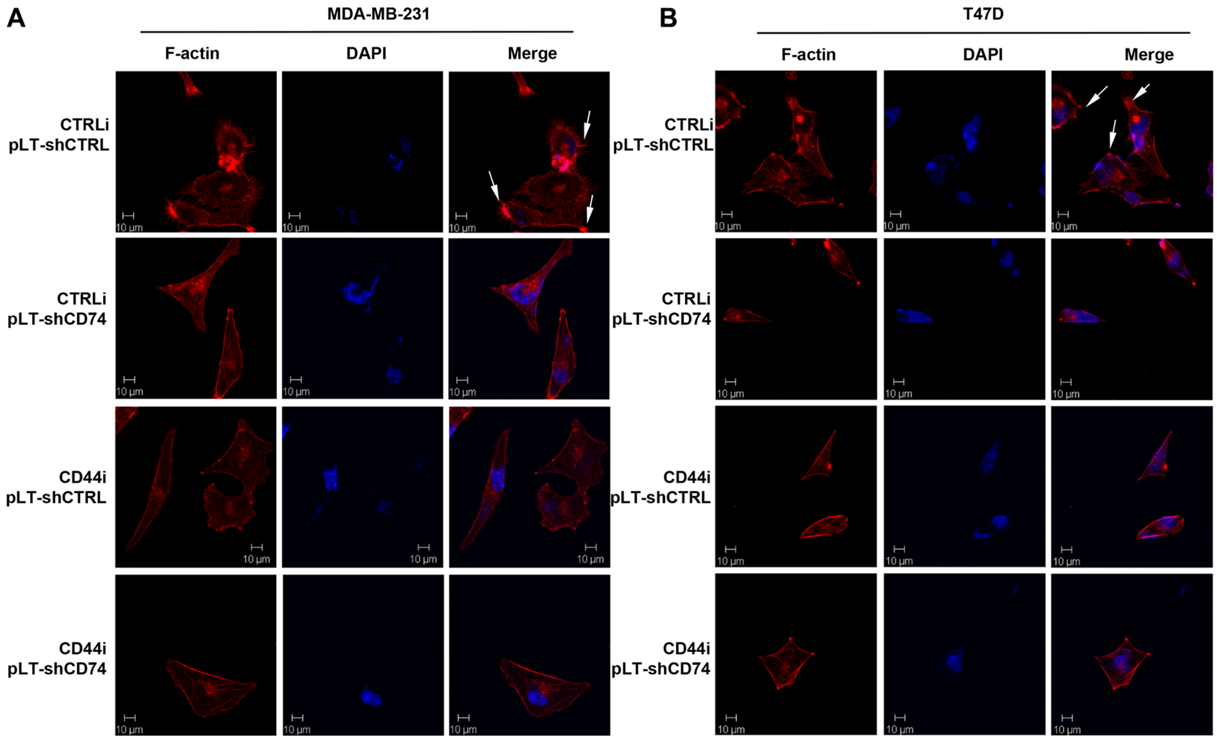

Supplementary Figure S3: Knockdown of CD74 or/and CD44 decreased cell protrusion formation. (**A**) Knockdown of CD74, CD44 or double knockdown of CD74 and CD44 were performed in MDA-MB-231 cells, the cells were resuspended and reseeded onto adhesion microscope slides after 24 h of transfection, F-actin was stained with TRITC-conjugated phalloidin (red), nuclei was visualized with DAPI (blue). Cell morphology was detected by confocal laser scanning microscopy. (**B**) T47D cells were treated similar to MDA-MB-231 cells, and representative images are shown as indicated. Arrows stand for the typical protrusions.

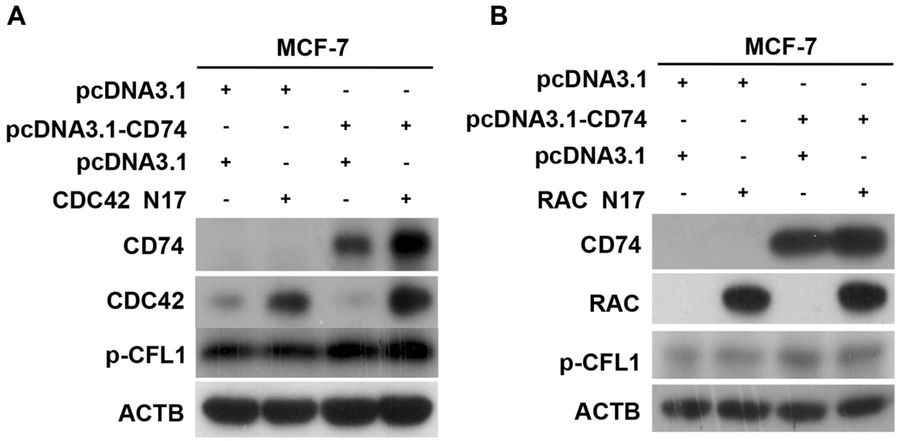

Supplementary Figure S4: Inhibition of CDC42 or RAC minimally down-regulated CFL1 phosphorylation. (**A**) MCF-7 cells were co-transfected with CD74 and CDC42 N17 (Dominant Negative), western blot analysis showed the phosphorylation of CFL1. (**B**) MCF-7 cells were co-transfected with CD74 and RAC N17 (Dominant Negative), CFL1 phosphorylation was assessed by western blot assay.

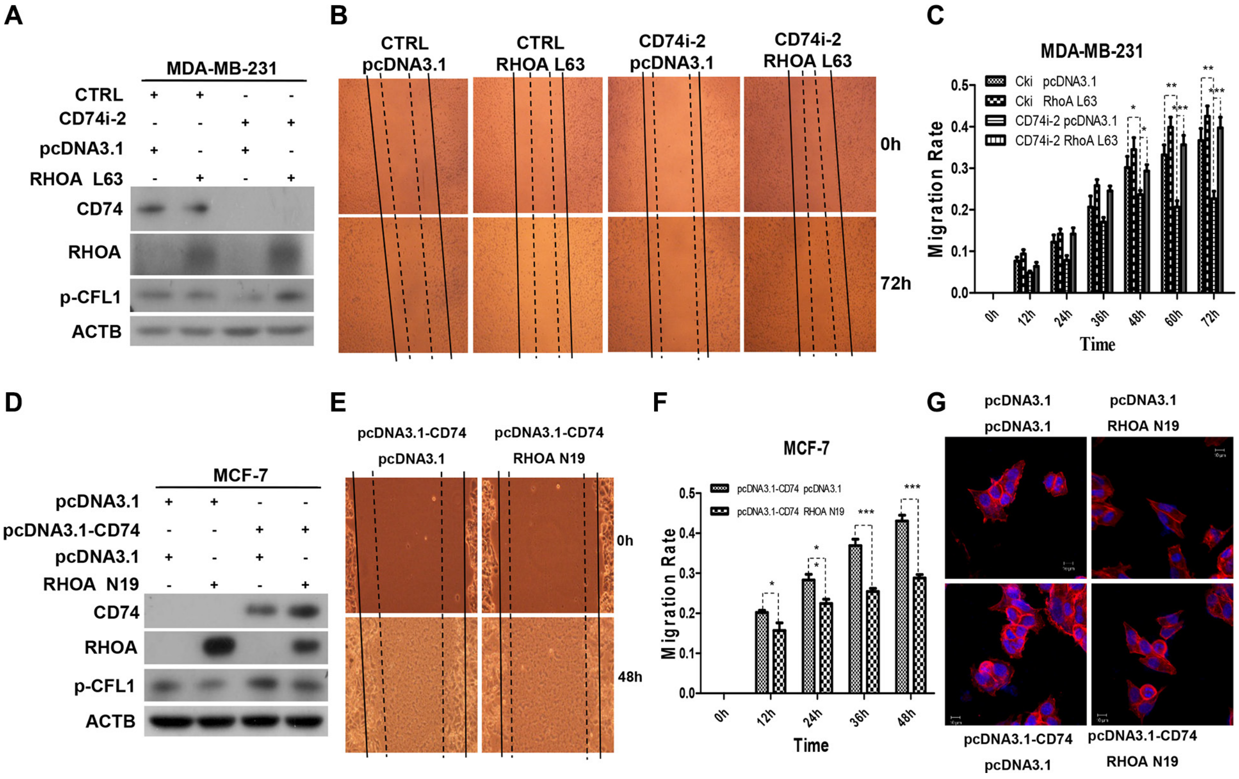

Supplementary Figure S5: RHOA is required for CD74 and p-CFL1-dependent cell migration. (**A**) MDA-MB-231 cells were co-transfected with CD74 shRNA contained plasmid and RHOA L63 (Constitutively Active) vector, RHOA-dependented CFL1 phosphorylation was analyed by western blotting. (**B**) MDA-MB-231 cells were subjected to wound-healing scratch assay after tranfecting as described above, cell were allowed to recover for 72 h. Representative images of cell recover are shown on the right. (**C**) Differences between groups were analyzed by *t*-test. The error bars represent the SD, **P* < 0.05; ***P* < 0.01; ****P* < 0.001. (**D**) MCF-7 cells were co-transfected with pcDNA3.1-CD74 and RHOA N19 (Dominant Negative) plasmids, RHOA regulated CFL1 phosphorylation was detected by immunoblot assay. (**E**) Wound-healing scratch assay was conducted after MCF-7 cotransfected with CD74 and RHOA N19. Cells were allowed to recover for 48 h. Representative images of cell recover are shown. (**F**) Differences between pcDNA3.1 plasmid transfected group and RHOA N19 plasmid transfected group were analyzed by *t*-test. The error bars represent the SD, **p*-value < 0.05 and ****p*-value < 0.001. (**G**) MCF-7 cells were co-transfected with CD74 and RHOA N19 for 24 h, then, re-suspended cells were re-seeded onto adhesion microscope slides, F-actin was stained with TRITC-conjugated phalloidin (red), nuclei was visualized with DAPI (blue).

Supplementary Table S1: 189 cases of BIDC patients' features

Patients characteristics	No. of patients (%)
Age (years)	
≤ 45	91 (48.1)
> 45	98 (51.9)
Clinical stages	
Stage I	9 (4.8)
Stage II	109 (57.7)
Stage III	50 (26.5)
Stage IV	21 (11.1)
Lymph node status	
N0	49 (26.0)
N1/N2/N3	140 (74.0)
Pathological grades	
Well	11 (5.8)
Moderate	106 (56.1)
Poor	72 (38.1)
Survival status	
Alive	144 (76.2)
Death	45 (23.8)

Supplementary Table S2: Comparison of CD74 protein expression in BIDC and non-cancerous control breast tissues

Features (*n*)	CD74		*P*-value
	Low expression (%)	High expression (%)	
Cancer (*n* = 189)	46 (24.3)	143 (75.7)	
NCBT(*n* = 40)	22 (55.0)	18 (45.0)	0.000*

*Chi-square test, statistically significant difference ($P < 0.05$). NCBT: non-cancerous control breast tissues.

Supplementary Table S3: Multivariate logistic regression analysis of lymph node metastasis factors in BIDC patients ($n = 189$)

Variables	B	S.E.	Wald	Sig.	Exp (B)	95.0% CI for Exp (B)	
						Lower	Upper
Clinical stages	1.863	.529	12.418	.000*	6.446	2.286	18.172
Pathologic grades	.067	.420	.025	.873	1.069	.469	2.438
Survival status	.255	.557	.210	.647	1.291	.433	3.848
ER	−.117	.880	.018	.894	.889	.158	4.989
PR	.372	.875	.181	.670	1.451	.261	8.064
CerbB-2	.175	.398	.193	.660	1.191	.546	2.596
Age(years)	.118	.360	.108	.742	1.126	.556	2.278
CD74	1.205	.412	8.573	.003*	3.337	1.490	7.478

95% CI: 95% confidence interval.*$P < 0.05$.

Supplementary Table S4: Analysis of the association between expression of CD74 protein and clinicopathological features of BIDC ($n = 189$)

Features	CD74		*P*-value
	Low expression (%)	High expression (%)	
Age (years)			
≤ 45 ($n = 91$)	21 (23.1)	70 (76.9)	
> 45 ($n = 98$)	25 (25.5)	73 (74.5)	0.697
Pathological grades			
Well/Moderate ($n = 117$)	30 (25.6)	87 (74.4)	
Poor ($n = 72$)	16 (22.2)	56 (77.8)	0.595
Clinical stages			
Stage I–II ($n = 118$)	23 (19.5)	95 (80.5)	
Stage III–IV ($n = 71$)	23 (32.4)	48 (67.6)	0.045*
Lymph node status			
LNM ($n = 140$)	28 (20.0)	112 (80.0)	
No LNM ($n = 49$)	18 (36.7)	31 (63.3)	0.019*
ER			
Negative ($n = 60$)	17 (28.3)	43 (71.7)	
Positive ($n = 129$)	29 (22.5)	100 (77.5)	0.383
PR			
Negative ($n = 65$)	19 (29.2)	46 (70.8)	
Positive ($n = 124$)	27 (21.8)	97 (78.2)	0.256
HER-2			
Negative ($n = 124$)	31 (25.0)	93 (75.0)	
Positive ($n = 65$)	15 (23.1)	50 (76.9)	0.770
Survival status			
Alive ($n = 144$)	33 (22.9)	111 (77.1)	
Death ($n = 45$)	13 (28.9)	32 (71.1)	0.415

*Chi-square test, statistically significant difference ($p < 0.05$). LNM: lymph node metastasis. *P* is used to test the association between expression of CD74 and the clinicopathological features of BIDC.
